# Acceleration of Healing in Full-Thickness Wound by Chitosan-Binding bFGF and Antimicrobial Peptide Modification Chitosan Membrane

**DOI:** 10.3389/fbioe.2022.878588

**Published:** 2022-04-25

**Authors:** Lin Hou, Wei Wang, Mei-Kun Wang, Xue-Song Song

**Affiliations:** Department of Anesthesiology, The First Hospital of Jilin University, Changchun, China

**Keywords:** CSBD-bFGF, antimicrobial peptides, chitosan, P5S9K, skin wound, skin dressing

## Abstract

Skin wound healing is an important clinical challenge, and the main treatment points are accelerating epidermal regeneration and preventing infection. Therefore, it is necessary to develop a wound dressing that can simultaneously cure bacterial infections and accelerate wound healing. Here, we report a multifunctional composite wound dressing loaded with chitosan (CS)-binding bFGF (CSBD-bFGF) and antimicrobial peptides (P5S9K). First, CS was used as the dressing matrix material, and P5S9K was encapsulated in CS. Then, CSBD-bFGF was designed by combining recombinant DNA technology and tyrosinase treatment and modified on the dressing material surface. The results show that the binding ability of CSBD-bFGF and CS was significantly improved compared with that of commercial bFGF, and CSBD-bFGF could be controllably released from the CS dressing. More importantly, the prepared dressing material showed excellent antibacterial activity *in vivo* and *in vitro* and could effectively inhibit the growth of *E. coli* and *S. aureus*. Using NIH3T3 cells as cellular models, the results showed that the CSBD-bFGF@CS/P5S9K composite dressing was a friendly material for cell growth. After cells were seeded on the composite dressing surface, collagen-1 (COL-1) and vascular endothelial growth factor (VEGF) genes expression in cells were significantly upregulated. Finally, the full-thickness wound of the rat dorsal model was applied to analyse the tissue repair ability of the composite dressing. The results showed that the composite dressing containing CSBD-bFGF and P5S9K had the strongest ability to repair skin wounds. Therefore, the CSBD-bFGF@CS/P5S9K composite dressing has good antibacterial and accelerated wound healing abilities and has good application prospects in the treatment of skin wounds.

## Introduction

Skin is the natural protective barrier of the human body, which can protect the body from physical and chemical damage and maintain the balance of the body’s internal environment (Martin, 1997). Once a large amount of skin is removed or destroyed, it will cause serious damage to the human body, such as amputation, infection, and death. Clinically, skin wounds are usually covered with dressings, which can inhibit bleeding and protect wounds from harmful environmental effects (Fleck and Simman, 2010; Jang et al., 2018; Vijayakumar et al., 2019). More importantly, wound dressings can not only provide a physical barrier for the wound but also maintain the moisture of the wound, absorb exudates from the wound, and promote wound healing. Thus, the selection of an appropriate wound dressing for different types of wounds is critical to wound healing. At present, gauze is the most commonly used dressing in the clinic, but it has many shortcomings, including frequent replacement, tissue adhesion and a lack of biological activity. With the development of biomaterials science, various synthetic or natural biomaterials have been utilized for the preparation of wound dressings. The ideal wound dressing material should be biocompatible, inexpensive, readily available, and highly antibacterial. Among all polymers being explored, chitosan (CS), a natural cationic polymer composed of N-glucosamine and N-acetylglucosamine, has attracted widespread attention in the field of wound treatment due to its unique biological characteristics (Wang et al., 2020). CS is considered an ideal wound dressing material because of its non-toxic, biodegradable, and antibacterial properties and its positive effect on skin tissue regeneration. Many studies have found that CS can promote wound closure and angiogenesis and reduce the risk of amputation (Chen et al., 2018; Bakhsheshi-Rad et al., 2020). Takei and his team demonstrated that CS dressing can accelerate skin tissue regeneration, reduce treatment frequency, and provide moist and comfortable skin wound protection (Takei et al., 2018). Furthermore, CS material also has excellent anti-inflammatory properties. It was found that the anti-inflammatory activity of CS was closely related to its molecular weight, and CS with high molecular weight (Mw ≤ 200 kDa) had better antioxidant and inhibition effect on the formation of inflammatory cells than CS with medium and low molecular weight (Mw ≤ 50 and 3 kDa) (Niu et al., 2021). CS wound dressings can regulate the secretion of various inflammatory mediators, such as interleukin-8, prostaglandin E, and interleukin-1 (Chou et al., 2003; Oryan et al., 2018).

In the treatment of skin wounds, an important treatment point is anti-infection. Open wounds easily cause bacterial invasion and growth, which can affect wound healing and even lead to serious complications such as systemic infection and limb necrosis (Miller, 1996). Therefore, a perfect wound dressing should have excellent antibacterial ability. Although CS material has a certain antibacterial ability, its ability is limited. One strategy to solve this problem is to add antimicrobial agents to dressing materials. At present, numerous antimicrobial chemicals have been applied to dressings to improve antibacterial activity and eliminate infection, among which antibiotics are one of the most widely used drugs (Rivero et al., 2020). However, common bacteria can become intractable antibiotic-resistant pathogens due to overuse of antibiotics, which can lead to the re-emergence of many deadly diseases. In the past few years, antimicrobial peptides (AMPs) have emerged as a new generation of antibacterial agents. AMPs are part of the innate immune system of all organisms and helps boost the body’s immunity against bacteria, fungi or viruses, and have a broad antibacterial activity spectrum, multiple modes of action and low cytotoxicity (Taute et al., 2019). More importantly, AMPs can greatly reduce the incidence of bacterial resistance compared with traditional antibiotics. Furthermore, AMPs have many advantages, such as being easily decomposed and eliminated by the human body, good thermal stability, small toxicity and side effects. Thus, in recent years, many researchers have used AMPs instead of traditional antibiotics to modify skin dressing materials and apply them to skin injury repair (Gomez-Sequeda et al., 2020). For example, based on optimizing the physicochemical properties of the peptide, Jenniffer and their team made reasonable modifications to its sequence and designed a new antibacterial peptide (P5S9K) (Cruz et al., 2018). The results showed that the AMPs adopted a helical structure in a simulated cell membrane environment and could pass through the cell membrane of pathogenic bacteria in a dose-dependent manner, thus reaching an antibacterial effect. More importantly, this antibacterial peptide is less toxic to eukaryotic cells. Therefore, using AMPs to modify CS dressings can effectively improve their antibacterial performance and accelerate wound healing.

In addition to bacterial infection, another important factor affecting wound healing is the reduction in wound environmental growth factor levels, which often leads to injury site ischemia and a slow tissue regeneration rate. Therefore, in the preparation of wound dressings, growth factors are often added to dressing materials to improve the tissue repair ability of wound dressing materials. Among all growth factors, basic fibroblast growth factor (bFGF) is one of the most frequently used and has received great attention in recent years (Xu et al., 2017). bFGF is a single chain polypeptide that can regulate various biological responses, such as cell growth, differentiation, and angiogenesis, which can promote acute and chronic wound healing and play a key role in the process of wound healing (Sun et al., 2014). Previous studies have shown that bFGF can stimulate the proliferation of multiple types of cells, such as fibroblasts and vascular endothelial cells, and promote the formation of granulation tissue, collagen deposition and skin tissue regeneration (Barrientos et al., 2008; Yan et al., 2015). However, the traditional blending method requires a high dose of growth factors to achieve the effect, which increases the cost of products. Furthermore, traditional biomaterials often have the defect of not being able to stably bind to growth factors, resulting in low retention of growth factors at the target site, exceeding physiological levels or having a short duration. Recently, some researchers introduced special binding domains into growth factors through DNA recombination technology and found that those recombinant growth factors have a strong binding ability to specific materials (Sun et al., 2007). The growth factors with specific binding domains could obviously improve the binding ability of polymer materials and growth factors so that the growth factor can be released in a controlled manner. Another advantage of this kind of growth factor is that the biological activity of the growth factor cannot be destroyed but also makes the polymer materials perform long-term tissue repair. For example, some studies prepared recombinant bFGF with collagen-anchoring ability to enhance the binding of bFGF and collagen scaffold, and the result indicated that this recombinant bFGF can obviously promote cell proliferation compared with commercial bFGF (Wu et al., 2018). (Zhang et al., 2019) introduced the DOPA domain into growth factor (IGF-1) *via* recombinant DNA technology and found that recombinant IGF-1 can be tightly bound to the surface of PLGA film (Zhang et al., 2019). More importantly, this recombinant IGF-1 can promote mesenchymal stem cells to secrete neurotrophic factors and provide a suitable cellular microenvironment for nerve cell growth. Thus, such growth factors with special material binding capabilities can not only reduce the amount of growth factor but also make the dressing materials perform long-term tissue repair ability.

**FIGURE 1:**
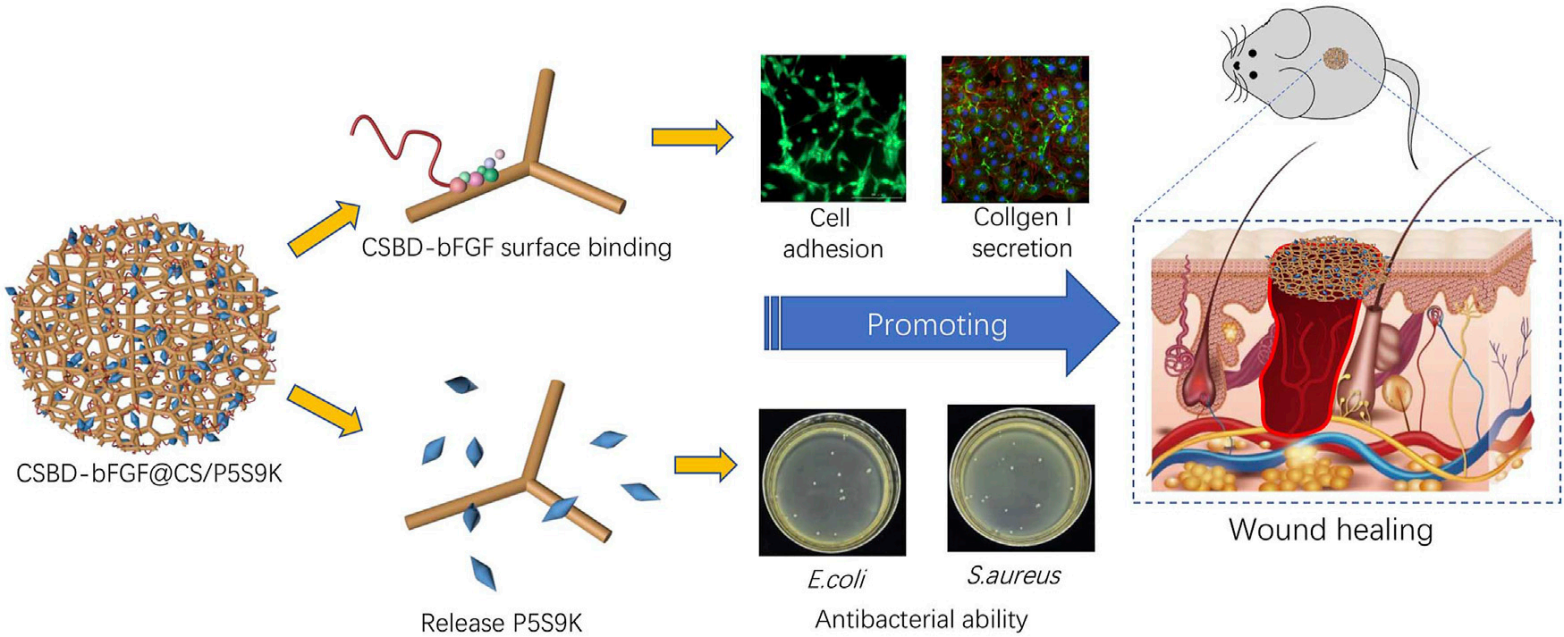
Preparation of the CSBD-bFGF@CS/P5S9K membrane and its effect on skin wound repair.

Based on the above findings, in this study, the antibacterial polypeptide P5S9K was added to the CS material to prepare composite membrane materials (CS/P5S9K) with excellent antibacterial ability. To further improve the tissue repair ability of dressing materials, the CS binding domain (CSBD) derived from chitinase A1 (ChiA1) was introduced into bFGF for the preparation of recombinant bFGF with CSBD, and CSBD-bFGF was then immobilized onto the CS/P5S9K dressing. The specific operation process is shown in Figure 1. The binding affinities of CSBD-bFGF and CS and the surface properties of the CSBD-bFGF@CS/P5S9K membrane were evaluated by ELISA assays, scanning electron microscopy (SEM) and contact angle tests. Meanwhile, the antibacterial properties of the CSBD-bFGF@CS/P5S9K membrane were evaluated *in vivo* and *in vitro*. The cell growth behavior of NIH3T3 cells was investigated by culturing on the CSBD-bFGF@CS/P5S9K membrane. Finally, we investigated whether the CSBD-bFGF@CS/P5S9K membrane can accelerate wound tissue regeneration in full-thickness wounds in a rat dorsal model.

## Materials and Methods

### Preparation of CS/P5S9K Membrane

The CS/P5S9K membrane was prepared *via* the solvent casting method. The 4% CS solution (W/V) was prepared by adding 2 g CS powder (deacetylation ≥95%, Mw ≤ 100 kDa, Aladdin, Shanghai, China) to 50 ml 1% acetic acid solution. Then, P5S9K (ATKKCGLFKILKGVGKI, synthesized by BGI Gene Co., Ltd.) was added. The final concentrations of P5S9K were 1, 2, and 3 mg/ml. Subsequently, the above mixture was stirred at 200 rpm overnight. Then, the solution was sonicated for 15 min and cast into a polystyrene dish. After vacuum drying for 24 h, the dry CS/P5S9K membrane was peeled off and immersed in 3% wt% NaOH solution for 10 min. Finally, this suspension was filtered and washed with distilled water. Then, the CS/P5S9K membrane was washed with distilled water and dried again. The fabricated CS membrane was dried and kept in a desiccator for future analysis.

### Preparation of CSBD-bFGF

The PET21b (CSBD-bFGF) plasmid was synthesized by BIG Co., Ltd. (Beijing, China), which included CS binding domain (TTNPGVSAWQVNTAYTAGQLVTYNGKTYKCLQPHTS LAGWEPSNVPALWQLQ) and bFGF gene sequence (154 aa). The PET21b (CSBD-bFGF) plasmid was transformed into the BL21 DE3 strain for subsequent protein expression. The strains were first cultured on a small scale for 12 h and then inoculated on a scale of 1:100 for large-scale culture. When the OD_600_ was 0.8, the recombinant protein was induced to be expressed with 0.2 mm isopropyl-β-d-thiogalactoside (IPTG) for 12 h at 25°C and 150 rpm. CSBD-bFGF recombinant proteins were prepared by Ni-NTA affinity chromatography and G25 desalination purification.

### Binding Efficiency and Release Profile of CSBD-bFGF and bFGF for the CS Membrane

The CS/P5S9K membranes (1 cm × 1 cm) were incubated in 1 ml CSBD-BFGF (500 ng/ml) and bFGF (500 ng/ml) at 4°C for 12 h. After protein adsorption, the membrane was washed with PBS. The two groups of membranes prepared by the above method were named CSBD-bFGF@CS/P5S9K and bFGF@CS/P5S9K, respectively. To evaluate the binding efficiency of the CS/P5S9K membrane to CSBD-bFGF and bFGF, CSBD-bFGF@CS/P5S9K, bFGF@CS/P5S9K and CS/P5S9K (Control) membranes were incubated with rabbit anti-human bFGF antibody (1:3,000 dilution) for 2 h at 37°C. Subsequently, all samples were washed three times with PBS and finally incubated with FITC-labelled goat anti-rabbit IgG antibody (diluted at 1:3,000) at 37°C for 2 h. Finally, fluorescence images were photographed and average signal intensity was analysed by a fluorescence imaging device (CRI Maestro). ELISA (R&D) kit was used to determine the amount of CSBD-BFGF and bFGF remaining in the protein incubation solution after protein absorption. The binding efficiency of CSBD-BFGF and bFGF with CS/P5S9K membrane was calculated by following formula:

Binding efficiency(%)=[500−A0]/500×100%

where A0 is the amount of protein remaining in the protein incubation solution.

CSBD-bFGF@CS/P5S9K and bFGF@CS/P5S9K membranes were incubated in PBS at 37°C for 40 h. At different time points, the growth factor content was measured by an ELISA (R&D) kit. The percentage of the cumulative content of released peptides and protein against time was calculated and drawn by Origin 8.0 software. All experiments were performed in triplicate.

### Antimicrobial Ability *In vivo* and *In vitro*

*Staphylococcus aureus (S. aureus)* and *Escherichia coli (E. coli)* were used as strains to evaluate the antibacterial performance of different samples. First, the concentration of the bacterial solution was adjusted to 3 × 10^4^ CFU/ml. After 2 h of culture in a 37°C constant temperature incubator, the bacterial solutions were cocultured with different concentrations of CS/P5S9K membranes (1 cm × 1 cm) for 12 h at 37°C. After that, the bacterial solution was diluted to a specific concentration, and 30 μl of diluted bacterial solution was spread onto the surface of LB agar medium. These plates were incubated for 20 h at 37°C. Finally, the culture plate was removed, and the colonies in the plate were photographed.

To evaluate the antibacterial activity of the CS/P5S9K membrane *in vivo*, different CS/P5S9K membranes (1, 2 and 3 mg/ml) were inserted into the back muscles of mice. Then, 100 µl of *E. coli and S. aureus* mixed bacterial solution with 10^6^ CFU bacteria/ml was added to the inside of the wound. After 4 days, the animals were killed, and the surrounding tissue containing membrane samples was removed. Finally, tissues were stained with hematoxylin and eosin (H&E) to observe the inflammatory response.

### Surface Characterization Analysis

After a conductive gold layer was coated on the surface of different membranes (CS, CS/P5S9K, bFGF@CS/P5S9K and CSBD-bFGF@CS/P5S9K), the surface morphology of the different membranes was observed by scanning electron microscopy (SEM, XL30 FEG, Philips). The surface chemical structures of the samples were characterized by FT-IR spectrometry (Tensor 27 FT-IR Bruker, Germany). The hydrophobicity and wettability of different membrane surfaces were measured by contact angle measurements. Two microliters of PBS was dripped onto different sample surfaces, and then the contact angle of each sample was detected by a drop shape analyser (DSA100, KRUSS, Germany). Finally, the average values of each sample were calculated.

### Cell Proliferation and Adhesion

*The in vitro* cell viability of the different samples (CS, CS/P5S9K, bFGF@CS/P5S9K and CSBD-bFGF@CS/P5S9K) was analysed using NIH3T3 cells (the Cell Culture Centre of Institute of Basic Medical Sciences Chinese Academy of Medical Sciences, Shang Hai, China). In brief, cells were cultured in DMEM (Gibco) with 10% FBS (Gibco), 1% penicillin and 1% streptomycin (Sigma) in a humidified incubator at 37°C with 5% CO_2_. Cell proliferation on different samples was determined by CCK-8 assay. The circular membrane with a diameter of 1 cm was sterilized with ultraviolet light for 30 min and then placed in a 24-well culture plate. Subsequently, 1 ml BALB/C 3T3 (3 × 10^4^ ml^−1^) cell suspension was seeded onto each membrane surface. After 3 and 7 days of culture, the medium was replaced by Cell Counting Kit-8 (CCK-8, Dojindo, Japan). At specific time points, the cell culture medium was removed, and CCK-8 solution was added to each well. After the cells were incubated for 2 h, 150 µl of solution in each well was transferred to a 96-well plate, and the samples were read at 450 nm by a spectrophotometric plate reader (Tecan Infinite M200). To visualize cell adhesion, the cells on different samples were first fixed with paraformaldehyde (4%) for 10 min. Then, FITC was used to stain the cell morphology. After the cells were washed with PBS, the morphology of the cells was observed by a fluorescence microscope (TE2000-U, Nikon).

### Tissue Repair-Related Gene Expression and Immunofluorescent

NIH3T3 cells were seeded on various membrane samples. At day 4, collagen-1 (COL-1) and vascular endothelial growth factor (VEGF) gene expression was detected *via* quantitative real-time polymerase chain reaction (qRT–PCR). The total RNA was extracted using TRIzol reagent (Beyotime, Shanghai) according to the manufacturer instruction. Then, the cDNA was synthesized using a PrimeScript^TM^ RT reagent kit (Takara, Japan) according to the manufacturer instruction. qRT–PCR was performed by Mx3005 (Stratagene, United States). The primers of repair-related genes are shown in Table 1, comprising GAPDH, COL-1, and VEGF. The PCR amplification cycles included denaturation for 5 s at 95°C, annealing, and extension for 34 s at 56°C for 40 cycles. The relative gene expression levels of COL-1 and VEGF were normalized to GAPDH.

**TABLE 1:** List of genes and primer nucleotide sequences.

Gene name	Forwards primer sequence (5′-3′)	Reverse primer sequence (5′-3′)
COL-I	CTG​AAA​TGT​CCC​ACC​AGC​C	GTC​CGA​TGT​TTC​CAG​TCT​GC
VEGF	CCTTCAGCTCGCTCCTCC	GAA​GAT​GAG​GAA​GGG​TAA​GCC
GAPDH	GAA​GAT​GAG​GAA​GGG​TAA​GCC	ACC​AGG​AAA​TGA​GCT​TGA​CA

At day 4, the cells were fixed with 4% paraformaldehyde for 10 min and then incubated with 0.25% Triton X-100 solution for 10 min. The cells were blocked with 10% goat serum for 30 min. Cells were incubated with polyclonal antibody to COL-1 (ABCAM, Shanghai) for 1 h. Next, the cells were incubated with Alexa Fluor® 488 labelled goat anti-rabbit IgG (ABCAM, Shanghai) antibody for 1 h. The cytoskeleton was stained with Alexa Fluor 555 labelled phalloidine (Beyotime, Shanghai) and the cell nucleus were stained with DAPI (Sigma, Shanghai). The immunofluorescent images were observed by a fluorescence microscope (TE2000-U, Nikon).

### *In vivo* Wound Healing Assessment

To further test the tissue repair ability of different membrane samples, the membrane was prepared into circular thin slices (diameter: 1 cm) and used to repair the full-thickness wounds of rats dorsal. Four groups were investigated: CS, CS/P5S9K, bFGF@CS/P5S9K and CSBD-bFGF@CS/P5S9K membranes. Twelve SD rats were anesthetized with 1% pentobarbital at a dose of 5 ml/kg, and the back hair of the rats was shaved. The exposed skin was sterilized with 75% ethanol, and then a circular wound 1 cm in diameter was cut by scissors. Finally, different membranes were implanted into the wound and fixed with gauze. After implantation at 0, 3, 6, 9 and 12 days, the wounds were observed by using a digital camera. Then, the wound area was measured by ImageJ software, and the wound closure rate was calculated as shown in the following formula:

Wound closure rate(%)=[A0−At/A0]×100%

A0 represents the initial area of the wound, and At represents the area of the wound on days 0, 3, 6, 9, and 12. All animal studies were approved by the Animal Ethics Committee of Jilin University, and all experiments were performed in accordance with relevant guidelines and regulations.

### Histological Evaluation

For histological examination, skin tissue was removed from the wound 12 days postsurgery. All specimens were fixed with 4% (w/v) paraformaldehyde. Then, the skin tissue was frozen and cut into 10 µm slices. Finally, H&E staining was performed in the histological analysis, and Masson’s trichrome staining was employed for collagen formation assessment. All samples were observed by a light microscope.

### Statistical Analysis

All experimental data were analysed by Origin 8.0 software (OriginLab, Los Angeles, CA, United States). Statistical differences between the data were assessed according to one-way analysis of variance, and all data are presented as the mean ± standard deviation (SD). *p* < 0.05 was considered statistically significant.

## Results and Discussion

### Bind and Release of CSBD-bFGF and bFGF on the CS Membrane

**FIGURE 2:**
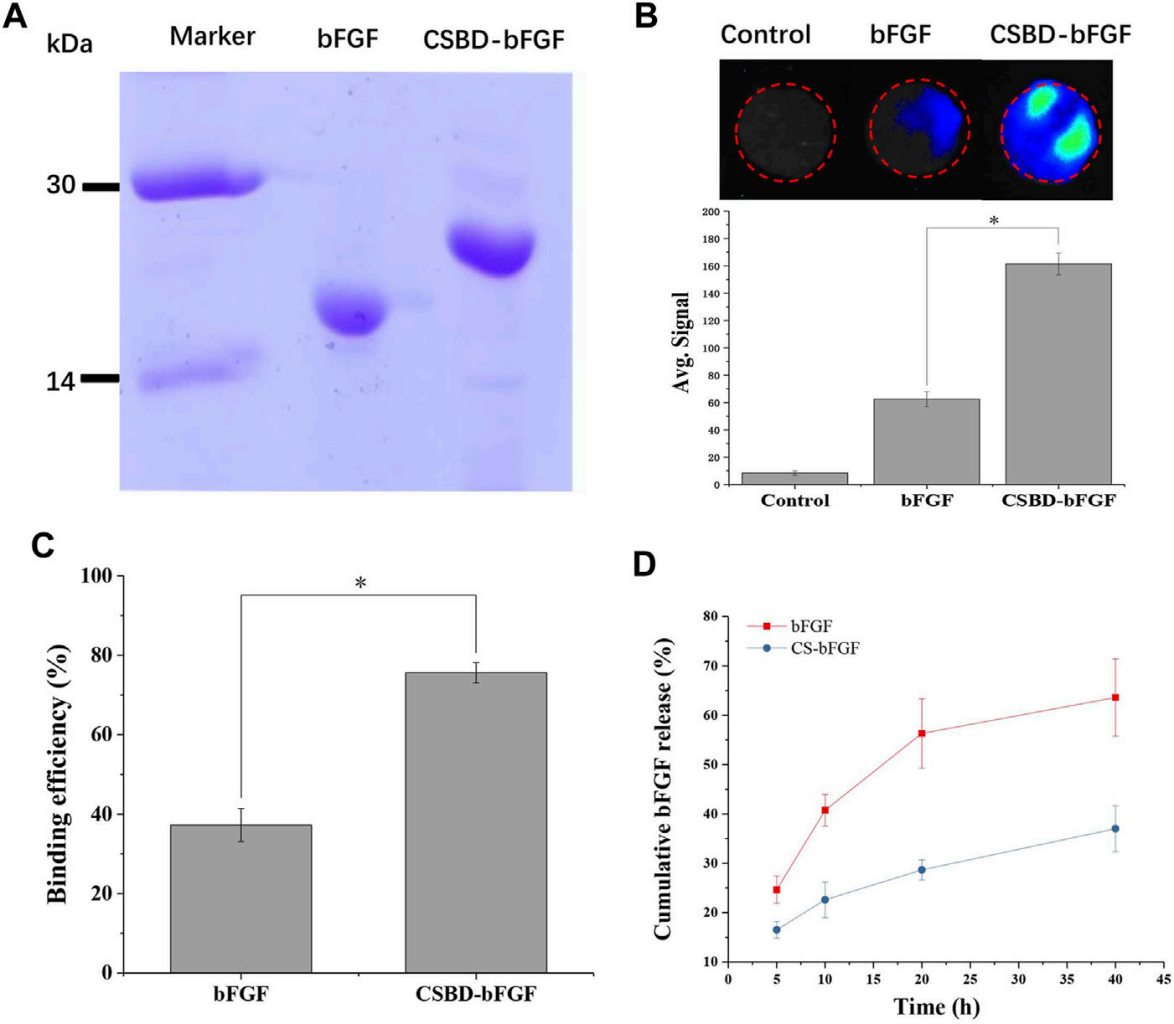
**(A)** SDS–PAGE of the bFGF and CSBD-bFGF fusion proteins, **(B)** immunofluorescence intensity represented the amount of bFGF and CSBD-bFGF, **(C)** binding efficiency and **(D)** cumulative release of bFGF and CSBD-bFGF, *p* < 0.05, *n* = 3.

As shown in Figure 2A, commercialized bFGF and recombinant CSBD-BFGF were detected by SDS–PAGE electrophoresis. The theoretical molecular weights of bFGF and CSBD-bFGF were 23.6 and 18.3 kDa, respectively. We could see that a single protein band appears in corresponding positions on the electrophoretogram, which indicated that CSBD-bFGF was successfully prepared.

When CS/bFGF composite dressing is used for wound treatment, the unstable combination of CS and bFGF often leads to the burst release of bFGF, and thus, high-dose bFGF is needed to improve the therapeutic effect. Large doses of bFGF can lead to many complications, such as cancer, angiogenesis, and vascular tumors (Zahra et al., 2021). Therefore, the main objective of this study was to enhance the binding ability of CS materials and bFGF and avoid the burst release of bFGF. To examine the CS-binding ability of recombinant growth factor, the CS membrane was incubated with CSBD-bFGF and bFGF. As shown in Figure 2B, the binding ability of the two growth factors to the CS membrane was detected using an immunofluorescence assay. The average signal intensity represented the amount of binding protein on the membrane. The results show that commercial bFGF had the lowest binding ability to the CS membrane due to the weak interactions of CS and growth factor. After CSBD was introduced into bFGF, the results indicated that the fluorescence intensity of the CSBD-bFGF@CS membrane was higher than that of the bFGF@CS membrane, and the average signal intensity of CSBD-bFGF was more than 2 times higher than that of commercial bFGF. As shown in Figure 2C, the binding efficiency of bFGF and CSBD-BFGF to CS membrane was 37.25% ± 4.16% and 75.58% ± 2.56%, respectively. Due to the introduction of CSBD binding domain sequence in bFGF, the binding efficiency of CSBD-bFGF and CS was improved by 38.33% compared with commercial bFGF. To further confirm our results, we studied the release profile of bFGF and CSBD-bFGF from the CS membrane for up to 40 h, as shown in Figure 2D. It is found that there are two stages in the release curve of bFGF and CSBD-bFGF: initial explosive release and subsequent stable release, which is the same as the release rule of most bioactive factors. The results showed that nearly 60% of bFGF was released from the bFGF@CS membrane in the first 20 h, and the total release of bFGF was approximately 65% in the following 40 h. However, in the CSBD-bFGF@CS sample, only approximately 30% of VEGF was released from the membrane in the first 20 h. Furthermore, the total release of CSBD-bFGF was significantly lower than that of bFGF in the following 20 h. Therefore, CSBD-bFGF can be retained on the CS membrane for a longer time and maintain a very slow release compared with bFGF, which effectively reduces the negative effects of bFGF burst release on skin wound repair. According to the above results, the design and synthesis of this recombinant bFGF can effectively overcome the shortcomings of growth factors and improve the binding ability of growth factors and CS materials.

### Antimicrobial Abilities *In vivo* and *In vitro*

**FIGURE 3:**
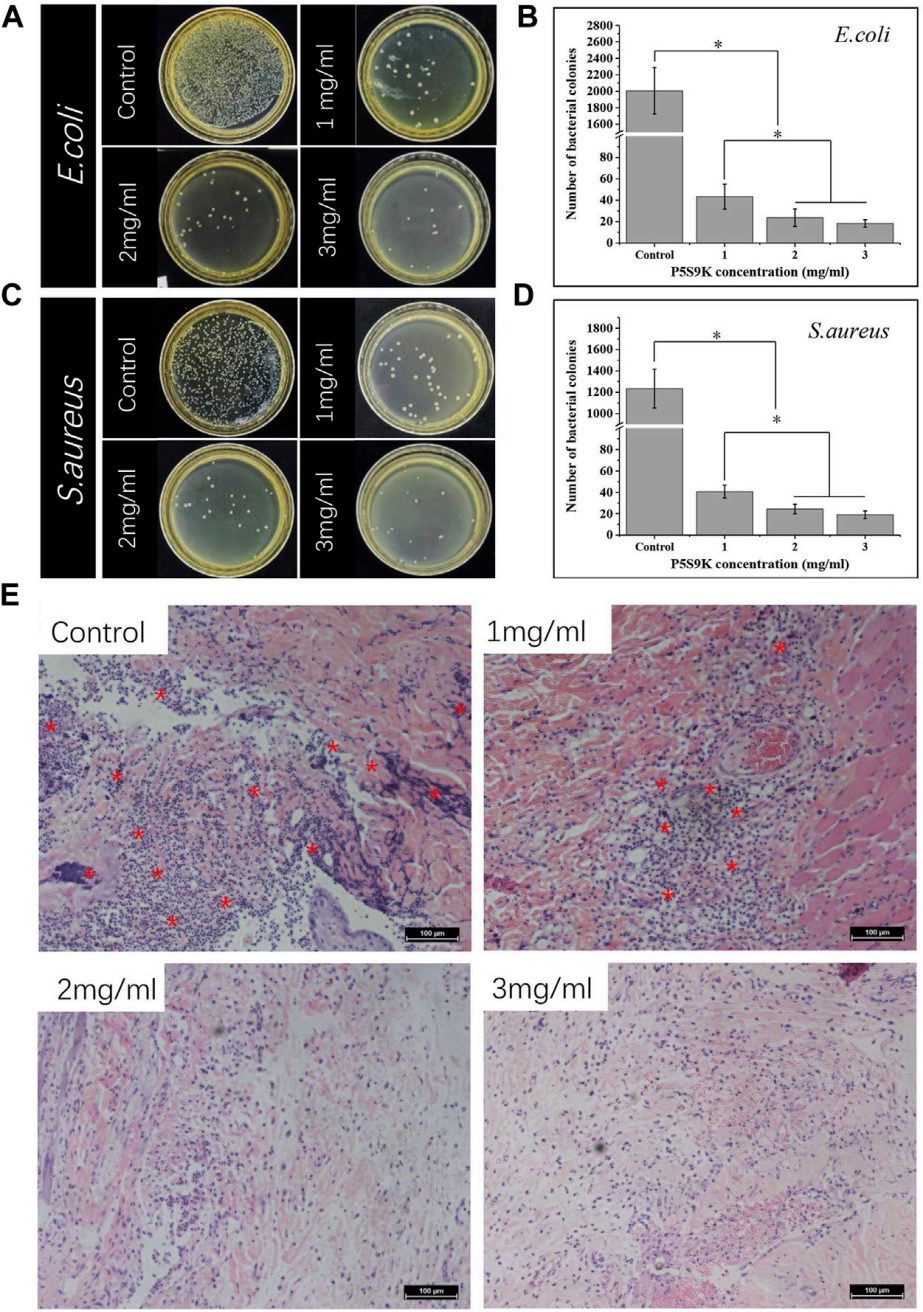
Photos and numbers of bacterial colonies formed by *E. coli* **(A,B)** and *S. aureus* **(C,D)** treated with different membranes (P5S9K: 1, 2, 3 mg/ml and CS: Control) **(E)** H&E staining of different membranes embedded in rat infected tissue. Red asterisks indicate areas of inflammatory cell infiltration, *n* = 3, *p* < 0.05.

In this study, to improve the antibacterial performance of the CS membrane and reduce the use of antibiotics, the antibacterial polypeptide P5S9K was added to the CS membrane. To verify the effect of AMPs on the antibacterial ability of CS membranes, CS membranes containing different concentrations of AMPs were prepared (1, 2 and 3 mg/ml). First, the bacteria were cocultured with different samples for 12 h, and then the bacteria were separated from the sample and planted into LB agar medium. The bacteriostatic effect of different samples was observed through the formation of colonies. As shown in Figures 3A–D, in the control group, the number of bacterial colonies was the largest, almost covering the entire culture plate. With the addition of P5S9K, the number of bacterial colonies in each group decreased sharply, and only a small number of bacterial colonies could be found in the culture plate. Subsequently, we evaluated the antibacterial activity of CS membranes containing different concentrations of P5S9K against bacterial-infected tissues. As shown in Figure 3E, H&E staining showed necrosis of surrounding tissues in the CS group, with diffuse lymphocytes, neutrophils, and macrophages. These results suggest that the presence of bacteria leads to a large amount of cell necrosis, and the antibacterial effect of CS is not obvious. After the addition of P5S9K, the necrotic area of the tissue decreased significantly, and the necrosis, nuclear fragmentation, and dissolution of muscle fibers decreased. Especially in the 2 mg/ml and 3 mg/ml groups, only a few inflammatory cells invaded the tissue, and the antibacterial effect of the material was the most obvious. Thus, the concentration of 2 mg/ml P5S9K was applied in the subsequent experiments. The above results indicated that P5S9K significantly improved the antibacterial activity of CS materials.

### Characterization of the CSBD-bFGF@CS membrane

**FIGURE 4:**
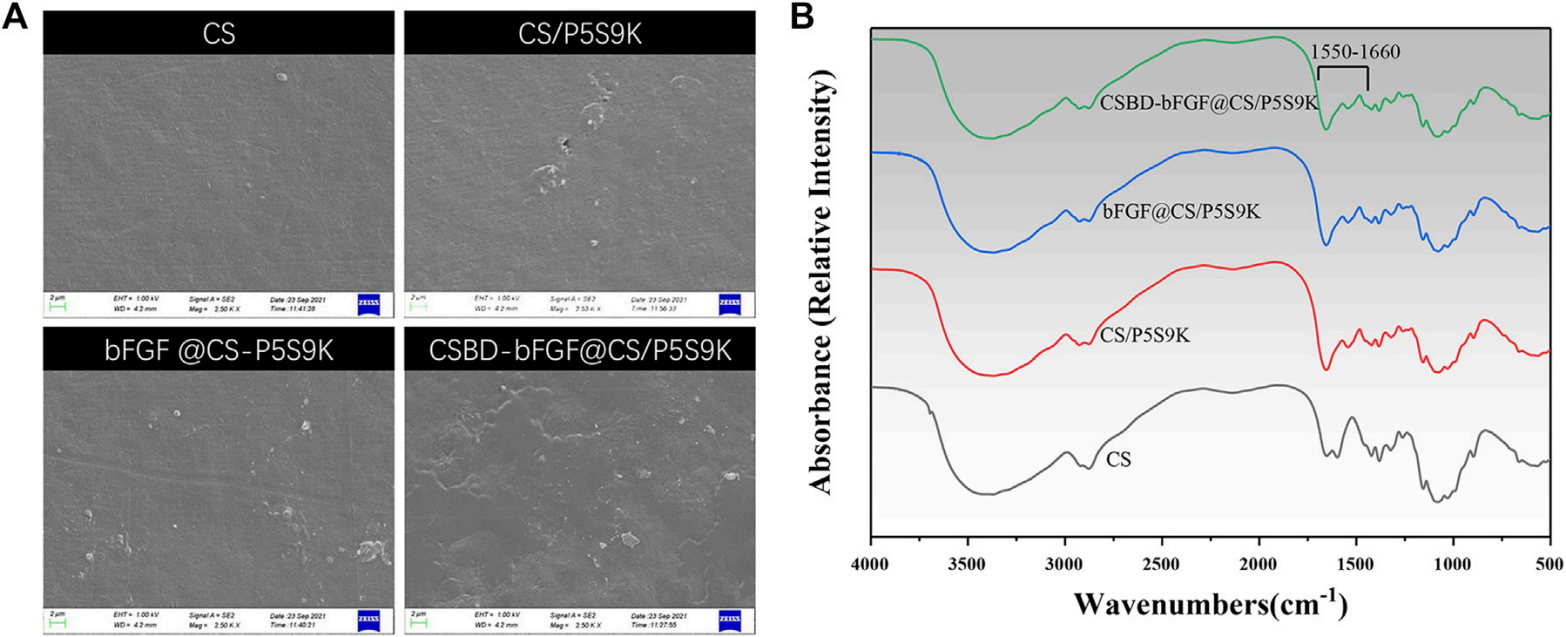
**(A)** SEM images and **(B)** FTIR spectra of different membranes. The scale bars represent 2 μm.

There are some necessary requirements for an ideal wound repair material, including hydrophilicity, antibacterial properties, transparency, and uniformity. Among these factors, the surface morphology of materials can affect their biological function directly or indirectly. Figure 4 shows SEM images of the surfaces of the CS, CS/P5S9K bFGF@CS/P5S9K and CSBD-bFGF@CS/P5S9K membranes. According to the SEM results, the pure CS membrane exhibit a smooth surface with a slightly textured structure. After P5S9K or bFGF binds to the CS membrane, the surface of the membrane becomes coarser than that of the CS membrane. Furthermore, compared with the bFGF@CS/P5S9K film, the surface roughness of the CSBD-bFGF@CS/P5S9K membrane is more obvious. This might be due to the strong interaction between CSBD-bFGF and CS, which led to more growth factor binding on the surface of the CS membrane. The above results show that the surface modification of CSBD-bFGF may affect the surface morphology of the materials. The FTIR spectra of the membrane were also obtained to determine the surface chemical composition of the samples. As shown in Figure 4B, compared to the spectrum of the pure CS membrane, a band at 1,550–1,660 cm^−1^ was observed, which represented peptide bonds derived from AMPs or growth factors.

**FIGURE 5:**
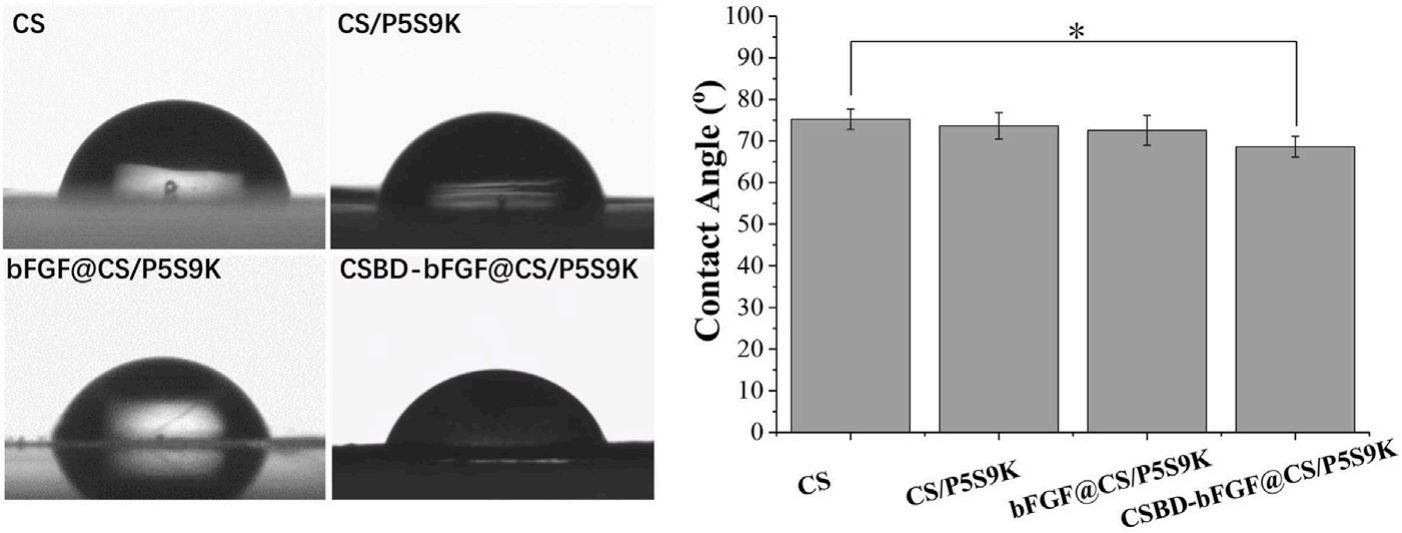
The water contact angles of the CS, CS/P5S9K, bFGF@CS/P5S9K and CSBD-bFGF@CS/P5S9K membranes, *n* = 5, *p* < 0.05.

The hydrophilicity of the material surface is one of the important parameters that affect the biological activity and repair effect of biomaterials (Ren et al., 2018). Compared with hydrophobic materials, cells can adhere and grow on the surface of hydrophilic materials (contact angle 30–90°) more easily. Furthermore, if the wound dressing materials are hydrophilic, they will keep the area around the wound moist, which will benefit wound healing (Hackl et al., 2014). In this study, the surface hydrophilicity or hydrophobicity of the different membranes was detected by means of contact angle analysis. As shown Figure 5, all membrane materials exhibit hydrophilic surfaces (*θ* < 90°). The CS films exhibited a contact angle of 75.2 ± 2.4° and were therefore relatively hydrophilic. After growth factor or antimicrobial peptides were bound to the membrane, there was a slight decrease in the contact angle of the membrane materials, and the contact angles of the CS/P5S9K, bFGF@CS/P5S9K and CSBD-bFG@CS/P5S9K membranes were 73.6 ± 3.2°, 72.5 ± 3.6° and 68.6 ± 2.5°, respectively. Compared with bFGF@CS/P5S9K, the water contact angle of the CSBD-bFGF@CS/P5S9K membrane decreases more obviously. We speculate that the presence of more hydrophilic protein on the surface of membrane materials is the reason for this observation. Furthermore, surface roughness also plays an important role in the contact angle. Based on the above results, this CSBD-bFGF@CS membrane has a very good hydrophilicity and can create a very good cellular microenvironment for wound healing.

### Cell Adhesion and Proliferation

**FIGURE 6:**
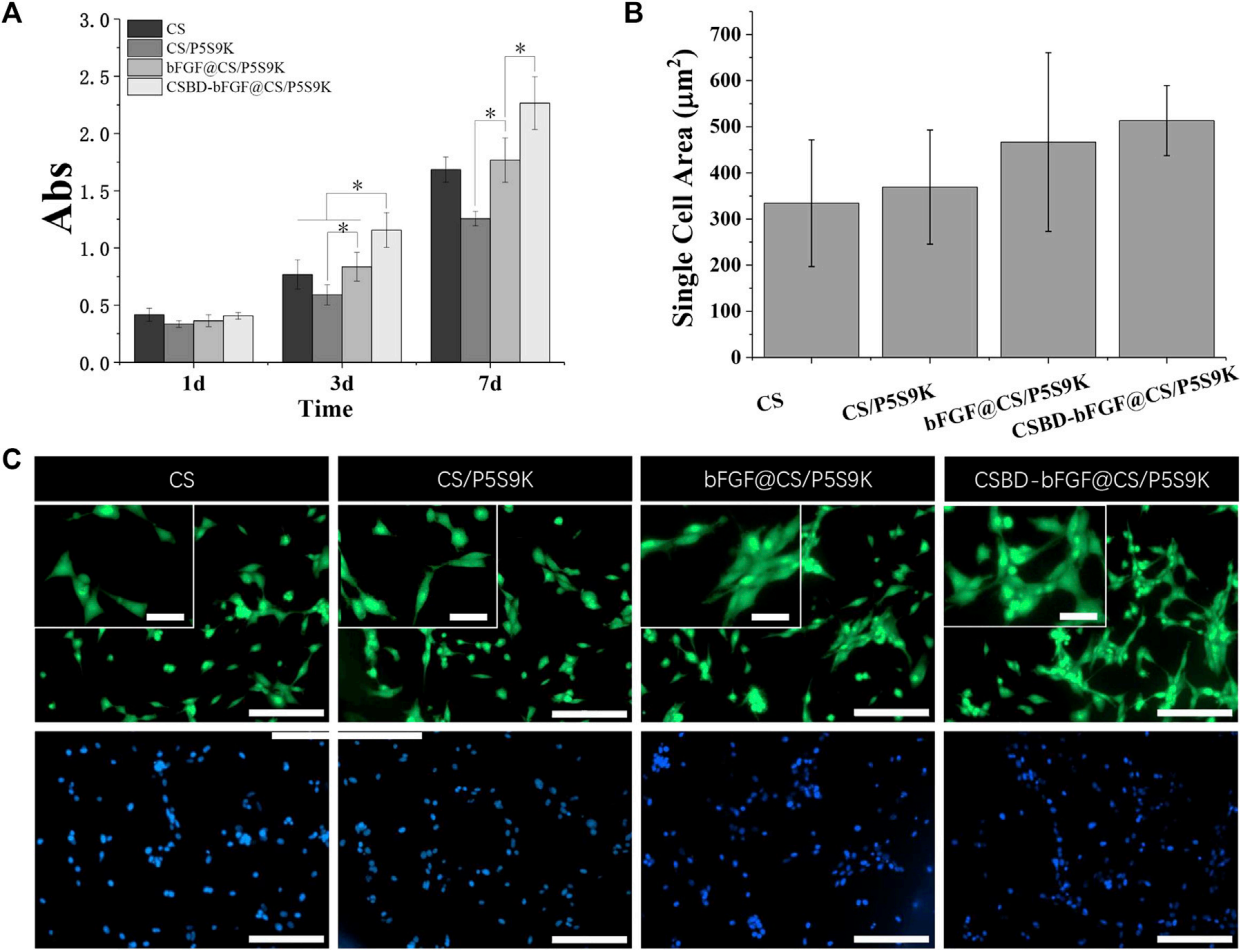
**(A)** CCK-8 test of NIH3T3 cell proliferation on CS, CS/P5S9K, bFGF@CS/P5S9K and CSBD-bFGF@CS/P5S9K membranes for 1, 3 and 7 days. **(B-C)** Fluorescent micrographs and cell area analysis of NIH3T3 cells on CS, CS/P5S9K, bFGF@CS/P5S9K and CSBD-bFGF@CS/P5S9K membranes for 2 days. The green fluorescent indicated the cell morphology stained with FITC, the blue fluorescent indicated the cell nucleus stained with DAPI. Small scale bar lengths are 50 μm, large scale bar lengths are 200 μm, *n* = 3, *p* < 0.05.

Fibroblasts are the major source of cells in new skin tissue and play a key role in skin tissue regeneration. In the early stage of wound healing, fibroblasts proliferate to form granulation tissue and secrete elastin, proteoglycan and hyaluronic acid (HA). Subsequently, the fibroblasts further differentiate into myofibroblasts with denser actin skeletons, which can promote wound contraction and ECM formation. When fibroblasts are cultured on wound dressing materials, the interaction with the materials may reveal the cellular compatibility of the wound dressing materials. In this study, NIH 3T3 cells were cultured on various membrane materials to detect the effect of recombinant bFGF and P5S9K on cell proliferation and adhesion. Cell proliferation on different membranes was investigated by CC-8 assay after 1, 3 and 7 days, as shown in Figure 6A. The results showed that NIH 3T3 cell viability increased over time in different membrane materials. Compared with the pure CS membrane, the OD values of cell viability on the CS/P5S9K membrane did not change significantly, indicating that P5S9K had no effect on cell viability. After adding bFGF, the proliferation ability of cells was significantly improved (*p* < 0.05). The results indicated that bFGF surface modification can obviously promote the cellular compatibility of the CS material, which is consistent with previous studies that have suggested that the promotion is due to the mitogenic and angiogenic characteristics of bFGF. After bFGF was fused with CSBD, the viability of NIH3T3 cells grown on the CSBD-bFGF@CS/P5S9K membrane was further improved at 3 and 7 days.

To further observe cell–cell interactions within the different membranes, the morphology and area of cells seeded on CS, CS/P5S9K, bFGF@CS/P5S9K and CSBD-bFGF@CS/P5S9K membranes was studied by FITC staining on day 2 Figures 6B, C, fluorescent imaging showed that NIH3T3 cells spread well on all membrane materials, and the morphology of cells on the membrane surface was normal with spindle shapes, suggesting that all membrane materials have excellent cellular compatibility. Compared to the CS and CS/P5S9K membranes, NIH3T3 cells more easily attached to the bFGF@CS membrane on day 2, and more cells were found on the surface of the bFGF@CS membrane. Furthermore, the largest number of cells was found on the surface of the CSBD-bFGF@CS/P5S9K membrane, the actin filaments of cells were interconnected more firmly, and the morphology of the adherent cells was larger in the CSBD-bFGF@CS membrane. The above results indicate that CSBD-bFGF can improve the surface performance of the CS membrane and promote cell growth, which was positively correlated with the CS binding ability of growth factor.

### Tissue Repair-Related Gene Expression

**FIGURE 7:**
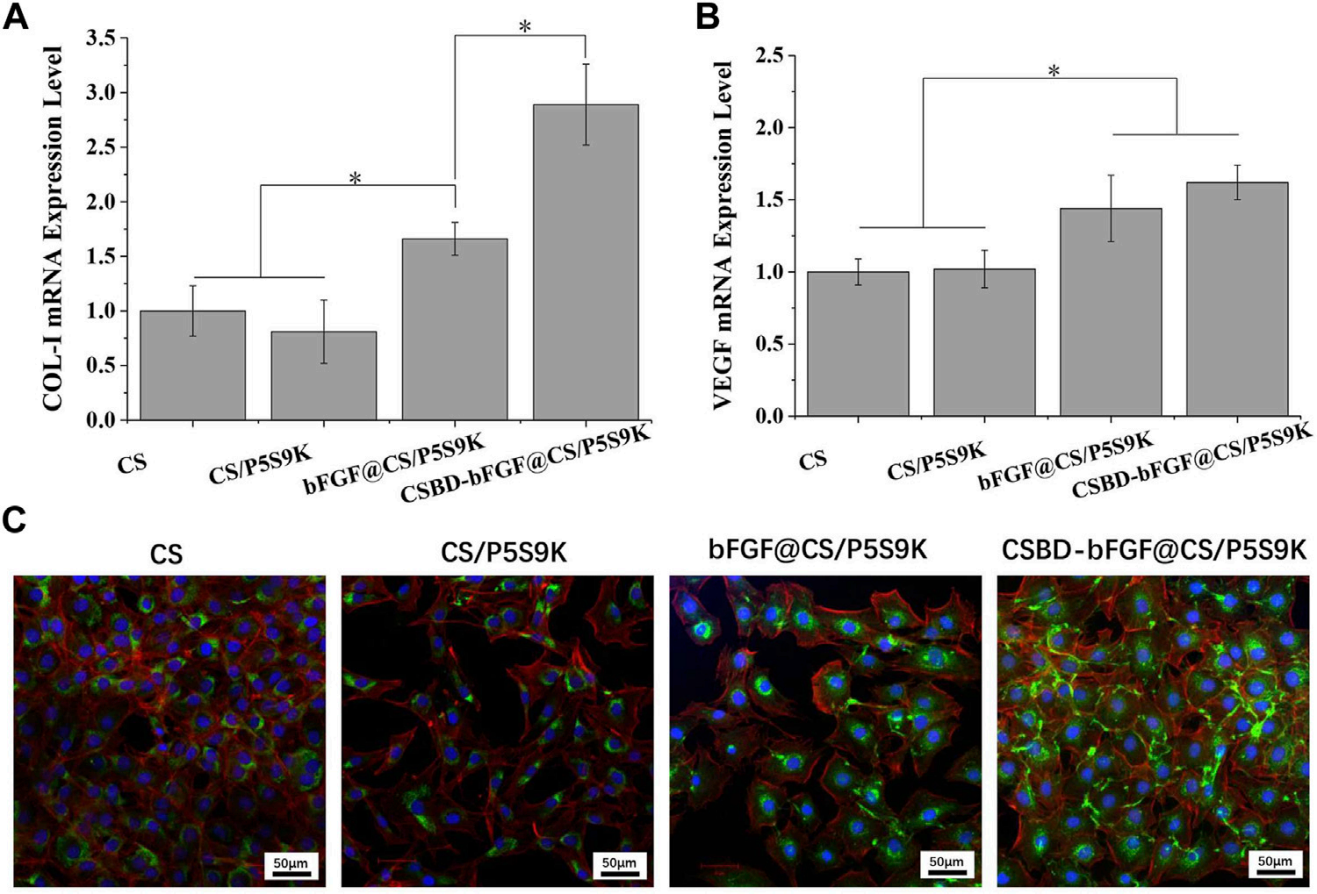
Quantitative real-time PCR analysis of tissue repair-related gene expression of **(A)** COL-I and **(B)** VEGF after NIH3T3 cells were cultured for 4 days. **(C)** Immunofluorescent images of COL-I protein for different samples on 4 days. The green fluorescent indicates the COL-I, the blue fluorescent indicates the cell nucleus stained with DAPI and the red fluorescent indicates the cytoskeleton stained with Alexa Fluor 555 labelled phalloidine. Scale bar lengths are 50 μm, *n* = 3, *p* < 0.05.

The main stages of cell growth on the biomaterial surface include cell adhesion, proliferation, differentiation and ECM formation, and these stages are regulated by different tissue repair-related genes (Hashimoto et al., 2020). COL-I is the main structural component of dermal tissue, and is higher in the dermis of normal skin, and COL-1 content can affects skin structure, function, and texture characteristics. VEGF is a major growth factor promoting neovascularization and can be as a marker for vessels and angiogenesis at the wound site, thus VEGF plays an important role in epidermal regeneration (McGettrick et al., 2009; Seo et al., 2018; Kim et al., 2021). Therefore, the expression of two tissue-related genes (COl-I and VEGF) was quantitatively analysed by real-time PCR, and the results are shown in Figure 7. For COL-I gene expression, the cells on the CSBD-bFGF@CS/P5S9K membrane showed higher mRNA expression levels of the target genes than those on the CS, CS/P5S9K and bFGF@CS/P5S9K membranes. CSBD-bFGF adsorbed on the surface of the CS membrane can maintain their biological activity and provide recognition sites for cell growth, so they can more effectively promote the expression of COL-I. For VEGF expression, the expression of VEGF in the CSBD-bFGF@CS/P5S9K group was also obviously higher than that in the other groups, which showed that VEGF mRNA expression was significantly upregulated in the CS membrane treated with CSBD-bFGF. However, the COL-I and VEGF expression levels of the CS and CS/P5S9K groups did not show any significant difference. These results indicate that the antibacterial polypeptide P5S9K has no significant effect on the expression of VEGF and COL-I in cells. Furthermore, CSBD-bFGF had a more significant effect on COL-I gene expression than VEGF expression. The above results suggested that the CSBD-bFGF@CS/P5S9K membrane was most effective at promoting the expression and translation of molecules related to tissue repair. This result further determined that CSBD-bFGF has a more positive effect on improving the tissue repair ability of skin wound dressings than commercial bFGF. The results of COL-I protein expression were further verified by confocal microscopy, as shown in Figure 7B. The area of COL-I staining around single cells in the CSBD-bFGF@CS/P5S9K group was significantly higher than that in the other groups.

### *In vivo* Wound Healing Assessment

**FIGURE 8:**
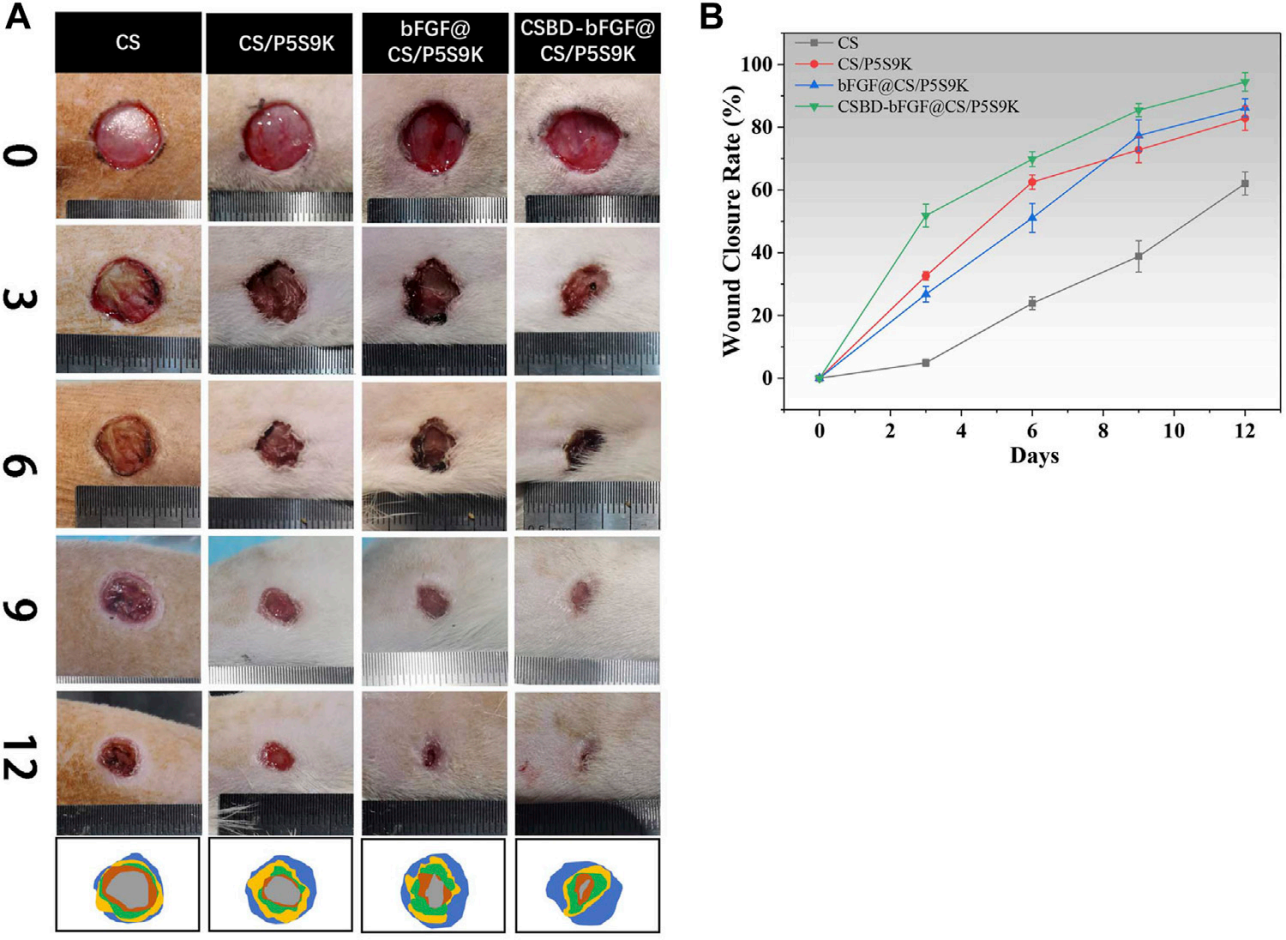
**(A)** Photographic images of the wound healing status and **(B)** the wound-healing curves.

An ideal wound dressing can create a good environment for skin regeneration and improve the speed of wound closure. Thus, the wound closure rate is an important index to evaluate wound healing conditions. As shown in Figure 8A, a rat full-thickness skin wound model was applied to evaluate the effect of the CS, CS/P5S9K, bFGF@CS/P5S9K and CSBD-CS-bFGF@CS/P5S9K membranes on the wound closure rate. The results showed the wound photos and healing rate of different membrane material treatment groups on days 0, 3, 7, 9 and 12. Over time, gradual decreases in the wound areas of all groups were observed. When the CS membrane contained P5S9K, the wound healing rate was significantly higher than that of the CS group. On day 12, the wound closure rate was 62.07% ± 3.69% in the CS group and 82.86% ± 3.87% in the CS/P5S9K group (Figure 8B). In the early inflammatory phase of wound repair, immune cells kill bacteria (Aviles et al., 2007). We speculate that P5S9K can effectively kill pathogenic bacteria, so the introduction of P5S9K into the CS membrane can shorten the inflammatory stage of wound healing. Furthermore, the wound area of the bFGF@CS/P5S9K and CSBD-bFGF@CS/P5S9K groups decreased significantly compared with that of the CS groups at 9 and 12 days. This result proves that bFGF can improve the tissue repair ability of wound dressings and promote wound healing speed. bFGF can promote angiogenesis by sending specific signals to different cell groups and the surrounding environment of the wound, thus promoting the efficient regeneration of tissues (Okumura et al., 1996; Akasaka et al., 2020). Furthermore, there was a significant change in wound area between the CSBD-bFGF@CS/P5S9K and bFGF@CS/P5S9K groups at 3, 6, 9 and 12 days. Compared with the CSBD-bFGF@CS/P5S9K group, the effect of the bFGF@CS/P5S9K membrane on wound regeneration was weaker, which may be caused by the unsatisfactory release of bFGF in the CS membrane. The quantitative results indicated that the wound closure rate of the CSBD-bFGF@CS/P5S9K groups was more than 85.42% ± 2.12% at 9 days and more than 94.43% ± 2.97% at 12 days, which was significantly higher than the wound closure rate of the bFGF@CS/P5S9K group. The above result reveals that the CSBD-bFGF@CS/P5S9K membrane can significantly promote skin wound closure.

### Histological Analysis

**FIGURE 9:**
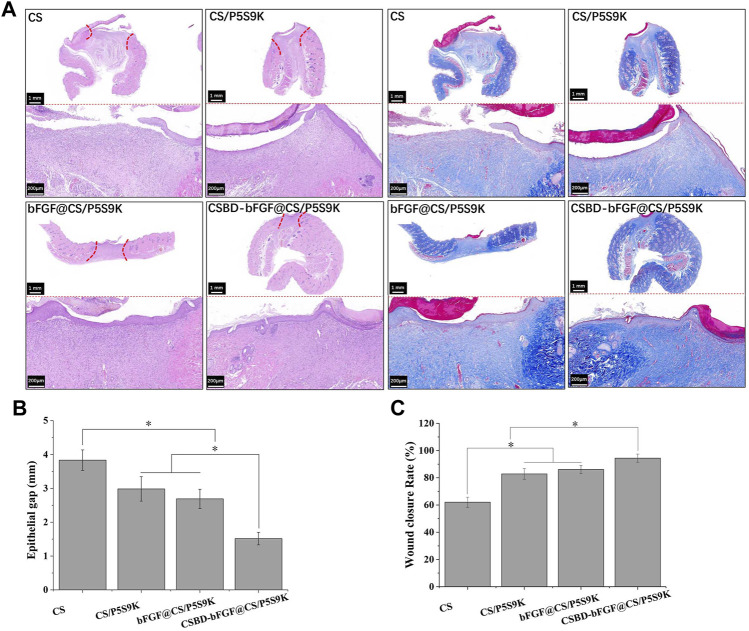
**(A)** H&E and Masson’s trichrome staining of the skin at day 12 post operation, **(B)** epithelial gap, **(C)** wound healing rate at day 12 post operation. Scale bars = 200 μm, *n* = 3, *p* < 0.05.

**FIGURE 10:**
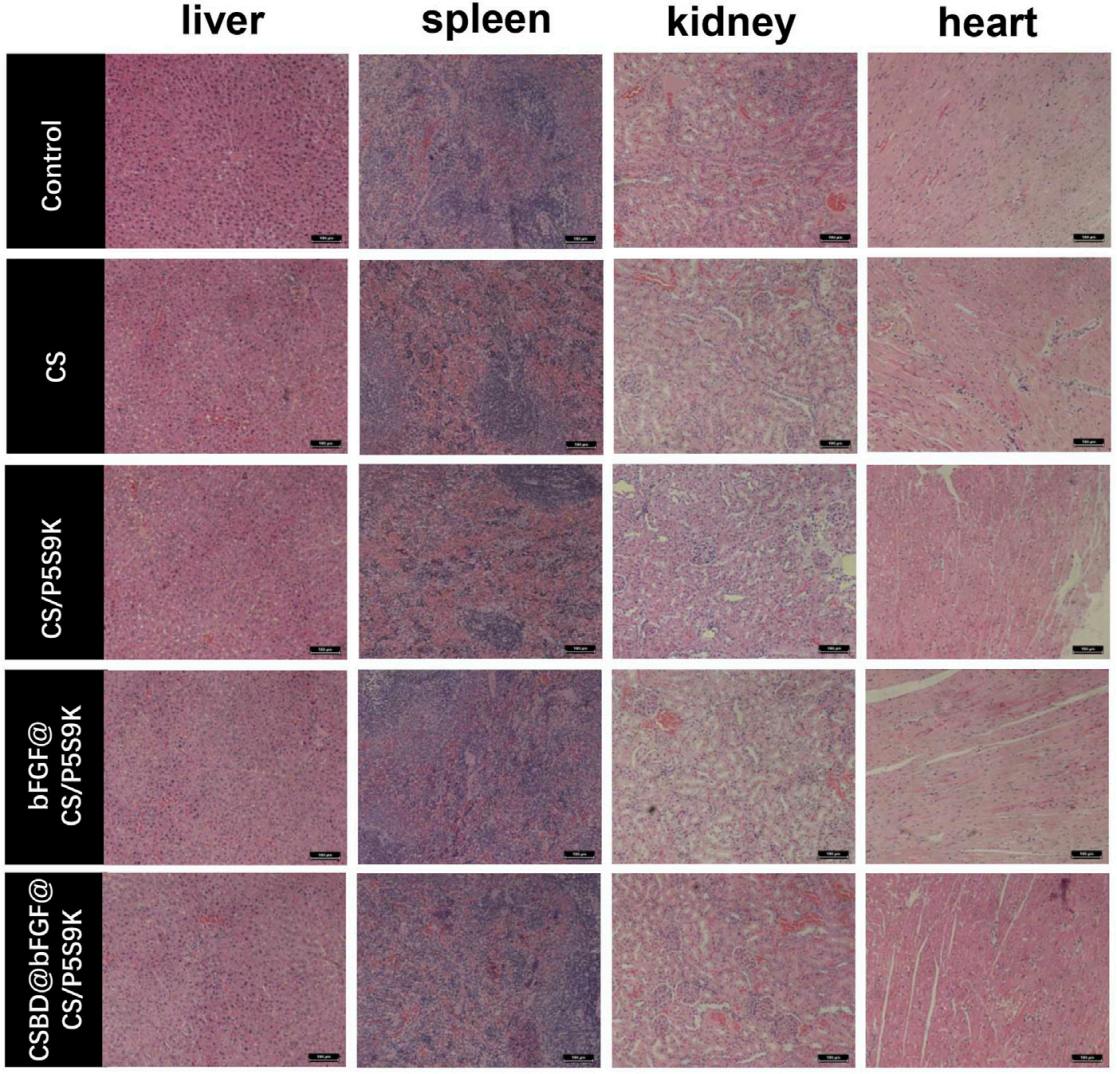
At day 12 post operation, H&E was used to stain sections of important organs (liver, spleen, kidney and heart) in experimental animals.

During the process of epidermal wound healing, fibroblasts proliferate on the wound surface and gradually differentiate into myofibroblasts and form granulation tissue, which eventually leads to wound contraction and collagen deposition. Furthermore, the keratinocytes on the wound surface proliferate and cover the wound to form a new epidermis, which is the re-epithelialization of the wound surface (Deshayes et al., 2018). To detect the effects of various membrane materials on re-epithelialization and collagen deposition, histological analysis of the epidermis of the repaired tissues was performed. As shown in Figure 9A, the wound closure in the CSBD-bFGF@CS/P5S9K group was the best, while wound closure in the CS group was the worst. The wound closure sequence was as follows: CSBD-bFGF@CS/P5S9K > bFGF@CS/P5S9K > CS/P5S9K > CS. The H&E results showed that the wounds of the CS group had a thicker new epidermal layer, and only a small amount of re-epithelialization and loose and disordered connective tissues were observed. Studies have shown that skin damage causes a temporary increase in epidermal thickness due to the proliferation and delayed differentiation of keratinocytes (Loh et al., 2018). Subsequently, the epidermal thickness gradually returns to normal with wound healing. Compared with the CS group, the new epidermis thickness of the CS/P5S9K and bFGF@CS/P5S9K groups was thinner, which indicates that the CS/P5S9K and bFGF@CS/P5S9K treatment groups were in the late stage of epidermal repair. After adding CSBD-BFGF, the thickness of the new epidermis was further reduced. Furthermore, there was a greater degree of granulation tissue formation in the CS-BS-bFGF@CS/P5S9K groups. Among all samples, the CSBD-bFGF@CS/P5S9K group had the best epidermal healing, it was found that the wound had basically repaired, and the basal cells were aligned. Collagen deposition on the wound surface was further evaluated by Masson trichrome staining. Collagen fibers in the CS group were irregularly arranged and sparse. Compared with the CS group, the collagen fibers of the CS/P5S9K and bFGF@CS/P5S9K groups were bunched, arranged in regular order, and closer to normal skin. In all treatment groups, the CSBD-bFGF@CS/P5S9K group had the highest collagen deposition, while the CS groups showed the lowest at 12 days. Consistent with the H&E results, the collagen deposition level of the CSBD-bFGF@CS/P5S9K group was obviously higher than that of the other groups, again demonstrating that the CSBD-bFGF@CS membrane is more effective in promoting skin wound regeneration. The epithelial gap and wound healing rate at 12 post operation were evaluated in Figure 9B,C, the CSBD-bFGF@CS/P5S9K group displayed the best wound repair ability. As an *in vivo* degradable material, the CS membrane could be absorbed and degraded *in vivo* after being wrapped by new skin tissue (Khan et al., 2020). Overall, all results show that the combined application of recombinant growth factors and AMPs can effectively enhance the tissue repair ability of CS membranes. Finally, to verify the biosafety of the different membrane samples prepared, membrane samples were implanted into rats. After 12 days, we removed the important organs of the experimental animals and observed the changes in the heart, liver, spleen and kidney through histopathology. As shown in Figure 10, compared with normal rats, the morphology and structure of visceral tissues in the implanted membrane materials group were normal, and no abnormal cells or structural changes were observed. Based on the above experimental results, the CSBD-bFGF@CS/P5S9K membrane prepared by us has good biocompatibility and tissue repair ability.

Extensive skin wounds caused by trauma, diabetes, and burns are common clinical diseases that have a negative impact on public health and significantly increase the cost of health care through increased infection, amputation, and mortality. Wound healing is a complex process involving angiogenesis, granulation tissue synthesis, re-epithelialization, and remodelling (Liang et al., 2021). These stages can be affected by factors such as infection or ischemia, which can slow the wound healing rate (Das and BakerMills and Sr, 2014; Das and Baker, 2016). Therefore, in many studies, various materials, such as collagen, CS, gelatin and synthetic polymers, have been used to deliver antibiotics or growth factors to skin wounds to accelerate the healing rate of skin wounds (Nurkesh et al., 2020; Kaiser et al., 2021; Prasathkumar and Sadhasivam, 2021). Some researchers developed CS scaffolds containing basic bFGF and treated the pressure ulcers of elderly mice, and results showed that CS/bFGF scaffolds significantly accelerated wound healing compared with pure CS group (Park et al., 2009). Other studies also found that loading EGF into CS materials can significantly accelerated the rate of epithelialization of injured skin (Hong et al., 2008). Amiri et al. (2020) successfully prepared CS-polyoxyethene nanofibers and loaded with antibiotics. The results showed that the CS-polyoxyethene nanofibers maintained the antibacterial activity of antibiotics, effectively inhibited bacterial growth and promoted skin wound healing. However, there are some defects in the use of traditional antibiotics or growth factors. For example, traditional antibiotics can increase the risk of bacterial resistance, while exogenous growth factors have disadvantages such as short half-life, easy degradability, low binding rate with wound repair materials, inability to retain their activity for a long time, and require continuous administration. Therefore, in this study, in view of the defects of antibacterial drugs and growth factors, the antibacterial peptide P5S9K was used to replace traditional antibiotics to reduce the risk of bacterial resistance. On this basis, we used bFGF and a CSBD to construct the recombinant growth factor CSBD-bFGF, which could specifically bind CS. The CSBD we chose in this study is derived from human fibronectin (it includes all the modules from I6 to I9) and has an excellent affinity for CS, which promotes very stable binding of recombinant growth factor to the CS membrane. The results showed that P5S9K could effectively improve the antibacterial activity of the CS membrane and inhibit the growth of *E. coli* and *S. aureus*. Furthermore, the binding capacity of the recombinant growth factor CSBD-bFGF was nearly three times higher than that of commercial bFGF. Through the release experiment, after a small amount of early sudden release, CSBD-bFGF can maintain a continuous and stable release within 40 h, which is the direct result of the formation of a stable complex between CS and CSBD. Subsequently, P5S9K and CSBD-bFGF were simultaneously applied to the functional modification of CS materials, and the tissue repair ability of CS was effectively improved, which significantly promoted cell proliferation, adhesion and tissue repair-related gene expression. *In vivo*, we studied and compared the effect of different membrane samples on rat wound healing. The results showed that the CSBD-bFGF@CS/P5S9K membrane significantly shortened the time of wound closure and reepithelialization. Compared with the other groups, collagen deposition was significantly increased in the CSBD-bFGF@CS/P5S9K group, and the appearance of new tissue in the CSBD-bFGF@CS/P5S9K group was similar to normal skin. The above results suggest that the CSBD-bFGF@CS/P5S9K membrane can effectively accelerate wound recovery and is a promising and effective wound dressing. We believe that this work can provide a valuable method for the application of growth factors and material surface modification and further expand its medical application as a wound dressing.

## Conclusion

In this study, we used the recombinant growth factor CSBD-BFGF and the antibacterial peptide P5S9K to enhance the biological function of CS material. The results showed that the antibacterial performance and tissue repair ability of the CS membrane were effectively improved by the double modification of recombinant growth factor and antibacterial polypeptide. More importantly, compared with commercial bFGF, the recombinant growth factor CSBD-BFGF had higher bioactivity and stability on the CS membrane and could be fixed on the CS membrane more effectively. *In vitro* and *in vivo* studies show that the CSBD-bFGF@CS/P5S9K membrane can effectively promote cell adhesion, proliferation, COL-1 and VGEF gene expression, and wound healing in rats. This might be due to the increased sustained and stable release of CSBD-bFGF promoting epithelial regeneration, angiogenesis and collagen deposition. Furthermore, the antimicrobial peptides effectively inhibited the growth of pathogenic bacteria, which can create a better cell microenvironment for tissue regeneration. Overall, our research work shows that using a functional CS membrane with CSBD-bFGF and P5S9K may be a promising and effective approach for skin wound repair applications.

## Data Availability

The original contributions presented in the study are included in the article/Supplementary Material, further inquiries can be directed to the corresponding author.
